# Die deutsche Abhängigkeit von russischem Gas: Ein Beispiel für Risikoblindheit bei politischen Entscheidungen

**DOI:** 10.1007/s10273-022-3224-2

**Published:** 2022-07-23

**Authors:** Werner Gleißner, Florian Follert

**Affiliations:** 1 FutureValue Group AG, Obere Gärten 18, 70771 Leinfelden-Echterdingen, Deutschland; 2 Fakultät für Management, Privatuniversität Schloss Seeburg, Seeburgstraße 8, 5201 Seekirchen am Wallersee, Österreich

**Keywords:** D72, D80, D81, M21

## Abstract

Risikomanagement erscheint zunächst als originär betriebswirtschaftliches Sujet. Globale Krisen wie die internationale Finanzkrise, die COVID-19-Pandemie oder der russische Angriffskrieg sowie die daraus folgenden energiepolitischen Fragen zeigen jedoch, dass der staatliche Umgang mit Risiken eine systematische Integration in den politischen Entscheidungsprozess benötigt. Es ist essenziell, Entscheidungstragende für staatliches Risikomanagement zu sensibilisieren und Instrumente sowie Institutionen aufzuzeigen, die in der Lage sein könnten, die gegenwärtige Risikoblindheit zu mindern, um Deutschland aus der Perspektive des Risikomanagements zu einem „robusten Staat“ weiterzuentwickeln.

## Abstract (en)

At first glance, risk management appears to be a topic original to business economics. However, global crises such as the international financial and economic crisis, the COVID-19 pandemic or the Russian war of aggression on Ukraine as well as the resulting energy policy issues show that governmental risk management needs to be systematically integrated into the political decision-making process. This paper aims to sensitise decision-makers in politics and society to the need for state risk management and to identify instruments and institutions that, taken together, could be capable of reducing the current “risk blindness” in order to further develop the Federal Republic of Germany into a “robust state” from a risk management perspective.

Zwischen 2008 und 2022 haben an sich bekannte volkswirtschaftliche Risiken zu Krisen geführt, die trotz aller Unterschiede (Gleißner und Kamaras, 2020) eine besorgniserregende Gemeinsamkeit aufweisen: Der deutsche Staat hat die Risiken nicht frühzeitig erkannt bzw. falsch bewertet, war auf diese nicht vorbereitet und daher mit dem Krisenmanagement überfordert. Auffallend ist, dass zwar kurzfristige Maßnahmen mit großem Eifer vorangetrieben werden, strukturelle Veränderungen auf einer übergeordneten Ebene als Lehren aus den Krisen (Daumann und Follert, 2021) jedoch kaum anzutreffen sind. Weder gibt es eine die verschiedenen Risiken priorisierende nationale Risikoanalyse noch ein strategisch angelegtes staatliches Risikomanagement, obwohl Sicherheit für Staat, Unternehmen und Bevölkerung als primäre Aufgabe des Staats anzusehen sind. Vor dem Hintergrund zahlreicher Interventionen in einzelnen Märkten ist es umso bemerkenswerter, dass der Staat offensichtlich bei allen Feldern, auf denen er sich inzwischen bewegt, seine originären Pflichten zunehmend vernachlässigt.

**Prof. Dr. Werner Gleißner** ist Vorstand der FutureValue Group AG, Leinfelden-Echterdingen und Honorarprofessor für Betriebswirtschaftslehre, insbesondere Risikomanagement, an der TU Dresden.

**Ass.-Prof. Dr. Florian Follert** ist Assistant Professor für Unternehmensrechnung und Sportökonomik an der Fakultät für Management der Privatuniversität Schloss Seeburg, Seekirchen am Wallersee, Österreich.

### Offensichtlich vernachlässigte Risiken

Trotz vorliegender Studien (Gleißner, 2020a; Deutscher Bundestag, 2013) war z. B. die Vorbereitung Deutschlands auf eine Pandemie nahezu nicht existent, wie schon das Fehlen von FFP2-Masken Anfang 2020 zeigte (Gleißner und Follert, 2021), und erforderliche Entscheidungen wie z. B. über die Impfstoffbeschaffung waren mangelhaft (Gleißner et al., 2021). Obwohl die Ambitionen der russischen Regierung nach Ausweitung der Einflusssphären der Russischen Föderation spätestens seit der Annexion der Krim im Jahr 2014 unübersehbar waren, gab es auch hier offensichtlich keine Vorbereitung auf eine mögliche Krise. Russland war und ist neben China (wegen Taiwan), dem Mittleren und Nahen Osten sowie der Kaschmirregion eines der besonders relevanten Felder für geopolitische Risiken. Wenn die Wahrscheinlichkeit steigt, dass Deutschland und die Europäische Union in einen gravierenden Konflikt mit Russland verwickelt werden könnten, müsste der Staat sich im Sinne eines adäquaten Risikomanagements frühzeitig darauf vorbereiten. Dies ist jedoch augenscheinlich nicht in hinreichendem Maße geschehen; ebenso wenig wie eine Vorbereitung auf einen möglichen Konflikt mit China, obwohl ein solcher im Extremfall mit einem vollständigen Stopp sämtlicher Importe aus China nach Europa enorme Auswirkungen hätte. Seit der erkennbaren Neuausrichtung der russischen Außenpolitik unter Präsident Putin ab 2008, spätestens jedoch ab 2014 wären Maßnahmen zur Bewältigung dieses zunehmenden geopolitischen Risikos erforderlich gewesen. Die Risikoforschung und die Methoden des Risikomanagements geben hier klare Orientierung (Blum, 2020). Bei einem möglichen Konflikt mit einem Staat wie Russland ist es offensichtlich notwendig,

- kritische Abhängigkeiten zu reduzieren,
- die Fähigkeit zu einer konventionellen militärischen Verteidigung sicherzustellen und
- sich auf mögliche Attacken unterhalb der Schwelle militärischer Maßnahmen einzurichten, z. B. auf Anschläge oder Cyberangriffe, die zum Zusammenbruch von Infrastruktur oder Wahlmanipulationen führen sollen.

Tatsächlich hat Deutschland keine adäquaten Maßnahmen zur Bewältigung des „Russland-Risikos" ergriffen; ebenso wenig wie die deutsche Bundesregierung systematisch die „Robustheit" des Staates im Hinblick auf andere denkbare Krisen verbessert hätte (Gleißner, 2020b). Erst nach dem Angriff Russlands auf die Ukraine wird zunehmend eingestanden – und in den Medien diskutiert –, dass die Bundeswehr zur Landesverteidigung de facto nicht fähig sei, eine gravierende Bedrohung durch Cyberangriffe bestünde und die Energieversorgung in Deutschland bei Haushalten und Unternehmen zumindest mittelfristig von den Lieferungen aus Russland abhängig sei.

Besonders erschreckend ist die Erkenntnis, dass die Bundesregierung in den vergangenen zehn Jahren das hier bestehende Risiko nicht etwa gemindert, sondern sogar erhöht hat. Entgegen den Nato-Vorgaben wurde einerseits zu wenig in die Bundeswehr investiert und andererseits die Abhängigkeit von russischem Gas erhöht. Durch die sogenannte Energiewende und den – als Entscheidung unter Kosten-Nutzen-Erwägungen kaum nachvollziehbaren – vorgezogenen Ausstieg aus der Nutzung der Kernenergie 2011 (Follert et al., 2021) hat sich die Abhängigkeit von russischem Gas erhöht.

Gleichzeitig wurden Alternativen wie etwa der risikodiversifizierende Bezug von Erdgas aus dem arabischen Raum zunehmend infrage gestellt. Da auf absehbare Zeit selbst bei weiterem Ausbau der erneuerbaren Energien keine adäquaten Speichermöglichkeiten zur Verfügung stehen dürften, benötigt man entweder grundlastfähige Kraftwerke oder flexibel nutzbare Kraftwerke, die bei Engpässen durch einen Mangel an Wind und Sonne schnell hinzugeschaltet werden können. In Deutschland wurde mit dem Ausstieg aus der Nutzung der Nuklearenergie und dem weltweit einzigartigen fast zeitgleichen Ausstieg aus der Stromproduktion durch Kohle ein Weg beschritten, der auf Jahrzehnte zur vollständigen Abhängigkeit von Gas führt, das im Wesentlichen günstig aus Russland importiert werden sollte. Die Angst vor den Risiken des Klimawandels hat offenbar eine adäquate Beurteilung der zunehmenden Abhängigkeit von Gaslieferungen aus Russland verhindert. Es lohnt sich zu erwähnen, dass die durch den Klimawandel bestehenden Risiken wie z. B. Extremwetterereignisse in Relation zu anderen Risiken zumindest auf absehbare Zeit für Deutschland durchaus überschaubar sind (Lomborg, 2022).

Es entsteht zunehmend der Eindruck, dass die Entscheidungen der politischen Akteure durch Risikoblindheit und das Ziel der kurzfristigen Wählerstimmenmaximierung durch einen Effekt geprägt sind, den man als „Zentralreduktion" bezeichnen kann (Dörner, 2003). Die öffentliche Wahrnehmung, die Wahlkampfthemen und damit letztlich auch die Politik sind durch wenige Themen geprägt – etwa den Klimawandel –, die sicherlich bedeutend sind, jedoch bei weitem nicht die einzigen relevanten Felder der Politik und alleinige Ursachen für Risiken darstellen. Nur so lässt sich erklären, dass sich Politiker:innen mit relevanten geopolitischen und volkswirtschaftlichen Risiken augenscheinlich nicht zur Genüge befasst haben und bei der Bewältigung der hier bestehenden Risiken grundlegenden Denkfehlern unterliegen, die im Vergleich zum durch gesetzliche Maßnahmen etablierten Risikogedanken in Unternehmen, einen ausreichenden Professionalisierungsgrad vermissen lassen.

## Der Soll-Zustand: der robuste Staat

Einen Denkrahmen für ein strategisches Risikomanagement bietet hier das Konzept des „robusten Staates" (Gleißner, 2020b), das

- ordnungspolitische Rahmenbedingungen für eine leistungsfähige Wirtschaft und moderate Verschuldung sowie hohe Bonität mit einem großen finanziellen Spielraum für eine Krise aufweist,
- systematisch „kritische Abhängigkeiten" identifiziert und zu vermeiden versucht,
- ausreichend Vorräte an Gütern vorhält, die in einer Krise besonders benötigt werden und nicht mehr in ausreichender Menge hergestellt werden können (von Energieträgern bis hin zu Ge- und Verbrauchsgütern des Gesundheitswesens),
- Bürgschaftsverbunde und Haftungsrisiken aus anderen Staaten eher vermeidet,

- durch quantitative Risikoanalysen und Krisen- bzw. Notfallpläne auf Extremrisiken und daraus folgende Krisen vorbereitet ist (von Pandemien über Lieferembargos bis hin zum Ausfall kritischer Infrastruktur, Blackout z. B. infolge von Cyberangriffen) und

- für die Krisenbewältigung notwendige, möglichst universell einsetzbare Ressourcen in angemessenem Umfang vorhält (wie z. B. im Gesundheitswesen und beim Militär; inklusive mobilisierbare Reserven aufgrund des recht flexiblen Fähigkeitsprofils z. B. im Bereich Sicherheit, Transport, Gesundheitswesen etc.).

Dass Deutschland dieser Lesart nach kein robuster Staat ist, ist offensichtlich geworden. Die bestehenden Schwächen bei der Robustheit, wie unzureichende Fähigkeit des Militärs oder das Ignorieren kritischer Abhängigkeiten, sind dabei wohlbekannt (Blum und Gleißner, 2021).

## Risikoanalyse, Risikostrategie und Political Judgment Rule

Die Chance dieser Krise besteht darin, dass Regierung und Bevölkerung durch die schnelle Abfolge von Krisen auf die Notwendigkeit eines strategischen Risikomanagements durch den Staat hingewiesen wurden. Aus den bekannten Methoden des Risikomanagements (Gleißner, 2022) lassen sich einige prima facie schlicht anmutende, aber für die Zukunft wichtige Botschaften ableiten.

### Erstellung einer nationalen Risikoanalyse

Ein sachgerecht priorisierter Einsatz knapper volkswirtschaftlicher Mittel, speziell Steuergelder, für die Bewältigung der vielfältigen Risiken, denen Deutschland ausgesetzt ist, setzt eine Risikoanalyse voraus. Dabei werden die wesentlichen Risiken zunächst identifiziert und dann quantifiziert. Letzteres bedeutet neben der Beurteilung der Eintrittswahrscheinlichkeit auch eine Quantifizierung der Bandbreite möglicher Auswirkungen. Auch wenn z. B. eine schwerwiegende Pandemie eintritt, deren Eintrittswahrscheinlichkeit bis 2019 auf 0,5 % pro Jahr geschätzt wurde, sind die Auswirkungen unsicher und durch eine Bandbreite zu beschreiben (z. B. der möglichen Letalität des Virus). Wir benötigen hier einen vorgegebenen Beurteilungsmaßstab, wobei bei den Risiken, denen ein Staat ausgesetzt ist, zwei Dimensionen betrachtet werden könnten:

- die monetären Auswirkungen (Verlust an Volkseinkommen bzw. Schäden des Volksvermögens) und

- die Auswirkungen auf die Menschen (z. B. Anzahl Todesfälle, Reduzierung der Lebenserwartung oder Disability-Adjusted Life Years (DALY)).

Konzepte des Abwägens beider Dimensionen sind aus der Gesundheitsökonomie bekannt (Gandjour, 2020; Daumann et al., 2022). Erst die Kenntnis des Beitrags von Einzelrisiken zum Gesamtrisikoumfang erlaubt eine Priorisierung von Risikobewältigungsmaßnahmen. Eine solche Risikoanalyse und der öffentliche Diskurs über die Ergebnisse sind nötig, um nicht die gesamte öffentliche Aufmerksamkeit und die Ressourcen des Staats sowie der Unternehmen auf wenige „Mode-Risiken“ zu lenken.

### Staatliche Risikostrategien auf dem Weg zu einem robusteren Staat

Die Kenntnis und Abschätzung der relevanten Risiken sowie ihre relative Bedeutung sind die Grundlage für die erforderliche Priorisierung von Maßnahmen und Ressourcen der Risikobewältigung. Eine Leitlinie hierfür bietet das Konzept des robusten Staats. Die Beachtung solcher Konzepte stellt sicher, dass z. B. die in einer Krise erforderlichen Ressourcen bevorratet und kritische Abhängigkeiten von einem nicht substituierbaren Lieferanten im Ausland vermieden werden. Ebenso lernt man, dass ein Staat und seine Bevölkerung grundsätzlich nicht für Schulden anderer Staaten haften sollten, deren Höhe sie nicht wirksam beeinflussen können.

### Implementierung einer Political Judgment Rule

Ebenso wie die Geschäftsleitung der Unternehmen sind bei allen Entscheidungen, die durch die Regierung bzw. das Parlament getroffen werden, die damit einhergehenden Risiken zu betrachten (Follert, 2018, 2020). Man benötigt hier eine Kosten-Nutzen-Kalkulation, die mit einer expliziten Betrachtung der mit der Entscheidung verbundenen Risiken einhergehen sollte. Auf Ebene der Aktiengesellschaft gibt es eine derartige Sorgfaltsverpflichtung. Aus betriebswirtschaftlicher Sicht sind verschiedene Handlungsoptionen nämlich mit unterschiedlichen Chancen und Gefahren verbunden, die im Rahmen der Entscheidungsfindung systematisch analysiert werden müssen. Es geht dabei nicht darum, politische Entscheidungen durch ein technokratisches Verfahren zu ersetzen. Geboten ist es aber, vor der eigentlichen Entscheidung eine „sachgerechte“ Entscheidungsvorbereitung zu implementieren und mit dieser sicherzustellen, dass insbesondere die Risiken im Entscheidungskalkül zumindest Berücksichtigung finden. Die Dokumentation der Annahmen, unter denen eine Entscheidung getroffen wird, zwingt das entscheidende Individuum zudem, sich intensiv mit potenziellen Handlungsfolgen auseinanderzusetzen (Follert, 2022). Man kann hier analog zur Business Judgment Rule der Aktiengesellschaft von einer Political Judgment Rule für die Verantwortlichen im Staat sprechen (Follert, 2018, 2020). Dieser Vorschlag wurde im Zusammenspiel mit einer Politikerhaftung von Follert (2018, 2020) eingeführt und aus rechtlicher Perspektive von Gebauer (2021) operationalisiert. Es mutet zunächst kurios an, dass die deutsche Gesetzgebung zu einer derartigen Entscheidungsvorbereitung zwar die deutschen Unternehmen, insbesondere die Vorstände von Aktiengesellschaften, verpflichtet, sich selbst aber keiner vergleichbaren Sorgfaltspflicht unterworfen hat. Bei Kenntnis des umfassenden Schrifttums der Neuen Politischen Ökonomie (stellvertretend Downs, 1957) lässt sich diese Asymmetrie jedoch leicht erklären. Die Einführung einer Political Judgment Rule in Verbindung mit der systematischen Risikoanalyse anstehender Entscheidungen ist hier zu empfehlen.

Eine solche Regel hätte dazu geführt, dass bei den Entscheidungen über die Energiewende, insbesondere nach dem abrupten Ausstieg aus der Nutzung von Nuklearenergie (Follert et al., 2021), die Implikationen der massiv zunehmenden Abhängigkeit von russischem Gas zumindest betrachtet worden wären. Studien über volkswirtschaftliche und geopolitische Risiken, Strategien und Instrumente für einen Wirtschaftskrieg und Methoden des Risikomanagements bieten insgesamt einen geeigneten Instrumentenkasten, um die hier skizzierten Methoden in der Realität auch umzusetzen.

## Hemmnisse auf dem Weg zum staatlichen Risikomanagement

Allerdings lehrt die Erfahrung mit dem oft unterentwickelten Risikomanagement deutscher Unternehmen eines: Ohne Sensibilisierung für die Bedeutung einer angemessenen „Risikokultur" werden die an sich verfügbaren und nützlichen Instrumente des Risikomanagements nicht genutzt. Dieses Problem besteht bei einem staatlichen Risikomanagement sogar noch eher als beim unternehmerischen Risikomanagement. Für die Verbesserung der Fähigkeiten Deutschlands im Umgang mit in Zukunft drohenden Krisen wie Blackouts, Zinskrisen oder dem möglichen Zerfall des Euros benötigt der robuste Staat ein höheres Risikobewusstsein. Dieses ist einerseits erforderlich auf Ebene von Regierenden und Parteien, aber ebenso auf der Ebene der Wählenden und der Medien, die Wahrnehmungsverzerrungen unterliegen können, was von opportunistischen politischen Akteuren zum eigenen Nutzen antizipiert werden kann (Follert und Daumann, 2020). Die Neue Politische Ökonomie arbeitet dezidiert heraus, dass die politischen Entscheidungen und das politische Angebot bei Wahlen wesentlich davon abhängen, was der entsprechenden Partei die meisten Wählerstimmen verspricht. Wenn nun der öffentliche Diskurs überwiegend von einem Risiko wie dem Klimawandel bestimmt wird, so ist es für die Parteien durchaus rational, sich eben auch nur mit Maßnahmen zur Bewältigung dieses Risikos zu befassen. Verfolgen Medien das Ziel der Informationsvermittlung, sind sie hier gefordert, bei ihrer Berichterstattung die tatsächliche Relevanz von Risiken zu betrachten – und nicht einfach immer nur über das zu berichten, was unabhängig von der Bedeutung besonders plakativ darstellbar ist (wie z. B. Naturkatastrophen, die die Medienberichterstattung stark bestimmen aber global insgesamt gerade einmal so viele Opfer fordern wie der Straßenverkehr in nur zwei Tagen – Terroranschläge sind eine noch viel geringere Bedrohung). Die verzerrte Medienberichterstattung gerade im Hinblick auf Risiken ist ein grundlegendes Problem, das für die Risikoblindheit der Regierung und damit die Vernachlässigung einer präventiven Beschäftigung mit relevanten Risiken mitverantwortlich ist. In dieser Hinsicht muss man kritisch anmerken, dass die negativen Auswirkungen z. B. des Ukrainekriegs infolge einer fehlenden Vorbereitung Deutschlands auch darauf zurückzuführen sind, dass die Politiker:innen gewählt wurden, die andere Prioritäten gesetzt haben.

Abschließend sei auf eine wichtige Limitation der Analyse hingewiesen: In unserem Plädoyer für eine Berücksichtigung der Erkenntnisse des Risikomanagements im politischen Entscheidungsprozess gehen wir überwiegend von eindeutigen und nicht konfligierenden Zielen aus. Dies ist eine starke Vereinfachung der Realität, in der sich Politiker:innen offenen Entscheidungsfeldern mit unbekannten Ursache-Wirkungsbeziehungen, Zielkonflikten und anderen Unvollkommenheiten gegenübersehen (Follert et al., 2021; Daumann et al., 2022). Grundsätzlich gilt dabei das Primat der Politik, sodass auch andere Maßstäbe als eine ökonomische Risikobewertung in das Entscheidungskalkül einfließen können. Entscheidend ist jedoch, dass entsprechende Erwägungen dokumentiert und begründet werden, damit politische Entscheidungen großer Tragweite, deren Handlungsfolgen die Bevölkerung treffen, ex post unter den im Entscheidungszeitpunkt vorliegenden Informationen intersubjektiv nachvollziehbar sind.

## Zusammenfassung

Wirtschaftliche und politische Krisen führen Politiker:innen und der Bevölkerung die allgegenwärtige Unsicherheit vor Augen. Vielen Gefährdungen kann a priori durch einen adäquaten Umgang mit Risiken begegnet werden. Was im unternehmerischen Kontext schon selbstverständlich sein sollte, erscheint im politischen Kontext etwa mit Blick auf die Abhängigkeit Deutschlands von russischen Rohstoffen als besonders verbesserungswürdig. Der vorliegende Beitrag plädiert daher für eine systematische Integration eines Risikomanagements in den politischen Entscheidungsprozess. Hierzu schlagen wir etablierte Instrumente wie quantitative Risikoanalysen vor, die durch eine Dokumentation von Entscheidungen inklusive der zum Entscheidungszeitpunkt gegebenen Informationen flankiert werden.

Der „Kampf“ gegen die Risikoblindheit ist auch auf gesamtgesellschaftlicher Ebene zu führen. Eine nationale Risikoanalyse und eine stärkere Institutionalisierung des Themas Risiko auf Ebene der Regierung, z. B. durch einen Risikobeirat analog dem „Ethikrat“, könnte hier Abhilfe schaffen. Aber die Erfahrung auf Ebene der Unternehmen zeigt, dass die von der psychologischen Forschung regelmäßig aufgezeigte Risikoblindheit – Risiken werden verdrängt, inadäquat priorisiert und bei Entscheidungen nicht hinreichend berücksichtigt – schwer zu bekämpfen ist. Die größte Chance ist daher die Sensibilisierung für die Bedeutung des Themas durch ein Risiko, das gerade eingetreten ist. Diese Chance haben wir nun!

**Title:** ***German Dependency on Russian Gas***
